# Design and Characterization of Spray-Dried Chitosan-Naltrexone Microspheres for Microneedle-Assisted Transdermal Delivery

**DOI:** 10.3390/pharmaceutics12060496

**Published:** 2020-05-29

**Authors:** Abayomi T. Ogunjimi, Jennifer Fiegel, Nicole K. Brogden

**Affiliations:** 1 Department of Pharmaceutical Sciences and Experimental Therapeutics, College of Pharmacy, University of Iowa, Iowa City, IA 52242, USA; abayomi-ogunjimi@uiowa.edu (A.T.O.); jennifer-fiegel@uiowa.edu (J.F.); 2 Department of Chemical and Biochemical Engineering, College of Engineering, University of Iowa, Iowa City, IA 52242, USA; 3 Department of Dermatology, University of Iowa Hospitals and Clinics, University of Iowa, Iowa City, IA 52242, USA

**Keywords:** microneedles, naltrexone, spray-drying, quality-by-design, transdermal delivery

## Abstract

Naltrexone (NTX) hydrochloride is a potent opioid antagonist with significant first-pass metabolism and notable untoward effects when administered orally or intramuscularly. Microneedle (MN)-assisted transdermal delivery is an attractive alternative that can improve therapeutic delivery to deeper skin layers. In this study, chitosan-NTX microspheres were developed via spray-drying, and their potential for transdermal NTX delivery in association with MN skin treatment was assessed. A quality-by-design approach was used to evaluate the impact of key input variables (chitosan molecular weight, concentration, chitosan-NTX ratio, and feed flow rate) on microsphere physical characteristics, encapsulation efficiency, and drug-loading capacity. Formulated microspheres had high encapsulation efficiencies (70–87%), with drug-loading capacities ranging from 10–43%. NTX flux through MN-treated skin was 11.6 ± 2.2 µg/cm^2^·h from chitosan-NTX microspheres, which was significantly higher than flux across intact skin. Combining MN-assisted delivery with the chitosan microsphere formulation enabled NTX delivery across the skin barrier, while controlling the dose released to the skin.

## 1. Introduction

The opioid epidemic is a major public health crisis in the United States, with over 72,000 overdose deaths recorded in 2017 and an estimated 2.1 million Americans (ages 12 and older) meeting the criteria for a diagnosis of opioid dependence [1]. Naltrexone (NTX) is a potent opioid antagonist administered orally and intramuscularly to manage opioid and alcohol dependence. Both routes of administration have significant disadvantages. Orally administered NTX undergoes extensive hepatic metabolism, can produce significant gastrointestinal upset, and results in large variations in plasma concentrations. Intramuscular administration is costly, requires medical expertise for administration, and may cause injection site pain; these concerns often lead to low patient compliance [2,3]. Transdermal delivery of NTX would overcome many of the challenges of oral and injectable delivery, as transdermal administration avoids first-pass hepatic metabolism, provides consistent drug plasma concentrations, and favors ease of application for patients [4]. However, the hydrophilicity of NTX, in addition to the strong barrier nature of the stratum corneum (the outermost layer of skin), limits the effective transdermal absorption of NTX [5,6]. These challenges make NTX an ideal drug candidate for an alternative transdermal delivery strategy that can improve its skin permeation, reduce side effects, and improve its ease of use.

Solid microneedles (MNs) have been demonstrated as a clinically useful method to facilitate transdermal NTX delivery [4,7]. As a minimally invasive delivery technique, MNs subtly pierce the skin and circumvent the stratum corneum. This results in the effective delivery of a broad range of therapeutics into deeper skin layers, including hydrophilic small molecular weight drugs, peptides, proteins, and other large molecules [8,9]. MNs act by creating aqueous micropores in the skin that serve as a transport route for therapeutics into deeper skin layers. More importantly, MNs are realistic for clinical implementation because they can be self-applied by patients and are generally well-tolerated [6,7,10,11].

When solid MNs are applied to the skin as a pretreatment to create micropores in the epidermis, the application of a drug formulation over the MN-treated area allows a drug to permeate through the micropores. Despite being a two-step process, this type of MN treatment affords some additional treatment flexibility when compared to other approaches such as coated or dissolving polymeric MNs. Higher doses can be administered using the two-step approach, which is appropriate for drugs like NTX. In vivo studies in humans have already shown that this MN technique effectively delivers NTX at therapeutic concentrations and is well-tolerated, with only minor NTX side effects such as nausea and lethargy reported [4]. Last, properties of the MNs (length and number) and the formulation (gel, solution, particles, etc.) can be adjusted, giving more flexibility in dosing. 

Microencapsulation is a method of producing particles containing active agent(s) that are surrounded by a network of polymeric membranes to form a capsule or matrix. The particles generally have a size between 1 and 1000 µm [12]. This technique has been used to entrap drugs into polymers, which are used to deliver high drug concentrations, protect the drug from degradation, and provide sustained drug release [13]. Biodegradable polymers, particularly natural polymers such as chitosan, have attracted more interest due to their affordability, biocompatibility, low irritation, little or no toxicity, and widespread availability [14]. Chitosan is a natural cationic linear polysaccharide that has been used extensively for drug delivery because of the aforementioned properties and, also, because of its mucoadhesive nature and ability for controlled drug release [15,16,17]. 

Spray-drying is a physical microencapsulation method of dry particle production through the rapid conversion of a fluid or slurry into dried powder by taking advantage of the gaseous hot-drying medium within the spray-dryer system. Spray-drying has found a wide range of applications in the pharmaceutical, food, pigment, and ceramics industries. The properties of the final products from a spray-drying process are often influenced by a variety of formulation and process parameters [13,18,19].

In this study, we used a quality-by-design (QbD) approach to design chitosan-NTX microspheres. Spray-drying was used to encapsulate NTX into chitosan microspheres because the technique is economical and can achieve a high drug-loading capacity. Using NTX as a model hydrophilic drug that is normally unable to permeate intact skin in clinically relevant amounts, we aimed to: (1) design and characterize spray-dried NTX-loaded chitosan microspheres, (2) quantify the influence of select formulation and process variables on microsphere characteristics, and (3) evaluate NTX in vitro skin permeation from the microspheres when delivered with MN skin pretreatments. 

## 2. Materials and Methods 

### 2.1. Materials

Naltrexone HCl was purchased from Mallinckrodt Pharmaceuticals (Webster Groves, MO, USA). Trifluoroacetic acid (TFA); triethylamine (TEA); HPLC grade water; acetonitrile; and low (50–190 kDa, 75–85% deacetylated), medium (190–310 kDa, 75–85% deacetylated), and high (310–375 kDa, > 75% deacetylated) molecular weight chitosan were purchased from Sigma Aldrich (St Louis, MO, USA). HEPES balanced salts, sodium bicarbonate, acetic acid, and SnakeSkin^®^ dialysis membrane (10,000 Da molecular weight cutoff) were purchased from Thermo Scientific (Watham, MA, USA). Porcine ear skin was purchased from a local slaughterhouse.

### 2.2. Design of Experiment (DoE)

To control chitosan-NTX microsphere quality attributes, critical process parameters that may affect the quality of the microspheres were selected based on a carefully researched knowledge space. Chitosan molecular weight (MW), polymer concentration, chitosan-NTX ratio, and spray-dryer feed flow rate were selected as critical formulation and process variables that may influence microsphere properties. Chitosan MW and concentration were selected because increased polymer MW and concentration have been shown to influence particle size and drug encapsulation efficiency [20], chitosan-NTX ratio was chosen as it impacts drug-loading capacity [21,22], and feed flow rate was selected for its proven influence on parameters such as yield and particle size in a spray-drying process [23]. Each variable was tested at 3 levels in a randomized 27-experiment Box Behnken design (Table 1), with variables at level −1, 0, and +1 depicting low, medium, and high levels, respectively (Table 2). Chitosan was used at low (50–190 kDa), medium (190–310 kDa), and high (310–375 kDa) MW; chitosan concentration was used at low (0.5% *w*/*v*), medium (0.7% *w*/*v*), and high (0.9% *w*/*v*) levels; chitosan:NTX ratio was used at low (1, one-part chitosan to one-part NTX), medium (3, three-parts chitosan to one-part NTX), and high (5, five-parts chitosan to one-part NTX); and feed flow rate was used at low (4 mL/min), medium (6 mL/min), and high (8 mL/min) levels. The influence of these variables on selected critical quality attributes (CQA) of chitosan-NTX microspheres were assessed using ANOVA analysis and general linear modeling to estimate parameters. The selected CQAs that described chitosan-NTX microspheres were yield, hydrodynamic diameter, zeta potential, encapsulation efficiency, drug-loading capacity, and percent cumulative NTX in vitro release. The inlet air temperature, atomizer flow rate, and aspirator pressure were kept constant at 190 °C, 750 mL/min, and 30 mbar, respectively; these values were efficient for spray-drying based on previous reports [23].

### 2.3. Spray-Drying of Chitosan-NTX Dispersions

#### 2.3.1. Chitosan-NTX Dispersion

Chitosan was dispersed in 1% *v*/*v* acetic acid under magnetic agitation for 24 h, the viscous chitosan dispersion was centrifuged at 10,000 x *g* for 10 min (Accuspin 400, Fisher Scientific, Hampton, NH, USA), and the supernatant containing dissolved chitosan was collected and used in the spray-drying process. The purpose of centrifuging was to remove any particulate material that may clog the spray-dryer atomizer nozzle. The amount of “undissolved” material seen after centrifugation was considerably low and likely had negligible effects on the product yield. NTX was dissolved in 20 mL of 1% *v*/*v* acetic acid and added to the dissolved chitosan dispersion to complete the volume for each batch ranging between 1.4 to 1.87 g of total dry weight. Before spray-drying, each batch was sonicated with a VCX probe sonicator (SONICS, Newtown, CT, USA) at an amplitude of 50 Hz for 2 min.

#### 2.3.2. Spray-Dried Chitosan-NTX Microspheres

Spray-drying was performed in a Buchi 190 mini spray dryer (Buchi Corporation, New Castle, DE, USA) equipped with a 1-mm nozzle (Niro Inc., Columbia, MD, USA) at an atomizer air flow rate of 750 mL/min, inlet temperature of 190 °C, and aspirator pressure of ~30 mbar [23]. Chitosan-NTX dispersions were pumped into the spray-dryer using a MasterFlex L/S peristaltic pump (Cole-Parmer, Vernon Hills, IL, USA) equipped with an Easy-Load II head and size 16 platinum-cured silicone tubing at feed flow rates set to values indicated in the experimental design for each batch. Outlet air temperature was recorded at the final minutes of completion for each batch. Dried chitosan-NTX microspheres were collected from the receiver chamber and weighed, and microsphere yield was calculated according to Equation (1):

(1) $$Yield\left(\%\right)=\frac{W_{r}}{W_{t}}x\;100$$

where *W_r_* is the total weight of chitosan-NTX microspheres recovered after spray-drying, and *W_t_* is the total weight of solid (chitosan + NTX) before spray-drying.

### 2.4. Characterization of Chitosan-NTX Microspheres

#### 2.4.1. Size Distribution and Morphology

Microsphere size distribution was determined by dynamic light scattering (DLS) using a Zetasizer Nano ZS (Malvern, Westborough, MA, USA). Chitosan-NTX microspheres were dispersed in absolute ethanol to prevent swelling and aggregation, while parameters of viscosity (1.061 cp), dielectric constant (24.3), and refractive index (1.361) for absolute ethanol [24,25,26] were adjusted on the instrument before analysis. Zeta potential was determined at 25 °C by laser doppler micro-electrophoresis technique.

Scanning electron microscopy (SEM) was performed to evaluate the microsphere shape and surface morphology using a Hitachi-S4800 microscope (Hitachi, Tokyo, Japan) at 5-kV acceleration voltage. Samples were prepared using double-coated carbon conductive tapes on stubs and were sputter-coated under vacuum before analysis with gold/platinum using an Emitech K550 sputter coater (Quorum Technologies Ltd, Laughton, UK). Fiji (ImageJ) software [27] was used to determine microsphere diameters of 100 randomly selected microspheres from the photomicrographs acquired with the SEM.

#### 2.4.2. Determination of NTX Content in Microspheres

Encapsulation efficiency was determined by dissolving a known weight of chitosan-NTX microspheres in 20 mL of 1% *v*/*v* acetic acid under magnetic stirring for 24 h. The solution was centrifuged (AccuSpin Micro 17R, Fisher Scientific, Hampton, NH, USA), syringe-filtered (0.45 µm, EMD Millipore, Burlington, MA, USA), and NTX content was quantified using a validated HPLC method, detailed later in the Methods section. Encapsulation efficiency was determined according to Equation (2), and drug loading capacity was determined according to Equation (3):

(2) $$Encapsulation\;efficiency=\frac{W_{experimental}}{W_{theoretical}}\times 100$$

(3) $$Drug\;loading\;capacity=\frac{NTX_{w}}{MS_{w}}\times 100$$

where W_experimental_ is the mass of NTX recovered from a known weight of chitosan-NTX microspheres, W_theoretical_ is the mass of NTX expected in an equivalent weight of chitosan-NTX microspheres, NTX_w_ is the mass of NTX recovered from a known weight of chitosan-NTX microspheres, and MS_w_ is the known weight of chitosan-NTX microspheres. Encapsulation efficiency and drug-loading capacity were determined in triplicate.

#### 2.4.3. Solid-State Characterization

Differential scanning calorimetry (DSC) measurements were performed on a Q_20_-DSC equipped with a refrigerated cooling system (TA Instruments, New Castle, DE). The DSC curves were obtained under nitrogen atmosphere (40 mLmin^−1^) using 1.5-mg samples and a heating rate of 5 °C·min^−1^. Accurately weighed samples (± 0.1 mg) were placed in a covered Tzero low-mass aluminum pan with an empty covered aluminum pan used as reference before heating from 0 to 240 °C.

FT-IR spectra were taken with a Frontier FT-IR/FIR spectrometer (PerkinElmer, Waltham, MA, USA). Approximately 5 mg of each sample was mixed and triturated with 300-mg KBr (dried overnight at 60 °C) in a mortar to form a fine powder mass. A small amount of the sample-KBr mixture was compressed for 2 min with a hand-press into a thin, translucent pellet disk and placed in the specimen holder of the spectrophotometer. All spectra were recorded at ambient temperature at a resolution of 1 cm^−1^ and 16-time scan. Each spectra scan was taken in the wavenumber region between 4000 to 400 cm^−1^.

### 2.5. In Vitro NTX Release Studies

In vitro release of NTX from the microspheres was determined using an in-line flow-through diffusion cell apparatus (PermeGear, Hellertown, PA, USA). HEPES-buffered Hank’s balanced salts (pH 7.4) with gentamicin sulphate (87 µM) served as the receiver medium. A cellulose dialysis membrane (SnakeSkin^®^, 10,000 Da molecular weight cutoff, Thermo Scientific, Waltham, MA, USA) was equilibrated in DI water for 10 min and then cut to fit the diffusion cells (2.2 × 2.2 cm internal dimension, with 0.64 cm^2^ permeation area). Chitosan-NTX microsphere dispersions containing 2-mg/mL NTX in PBS (pH 7.4) were prepared, and 500 µL was applied to the dialysis membrane. Dispersions were prepared by adding the required weight of chitosan-NTX microspheres into the dispersion medium under magnetic agitation before they were applied to the donor chamber. The diffusion cells were maintained at 37 °C throughout the study, and the receiver medium flow rate was set to 1.5 mL/h to maintain sink conditions. Samples were collected every 3 h for 24 h. Cumulative NTX release was quantified using HPLC. Experiments were performed in triplicate.

### 2.6. In Vitro Skin Permeation Studies

#### 2.6.1. Preparation of Chitosan-NTX Dispersions

Chitosan-NTX dispersions of three formulations (F7, F19, and F27 (Table 1)) containing 12-mg/mL NTX were prepared by dispersing the necessary mass of each formulation in a dispersion medium composed of DI water (79%), polyethylene glycol (20%), and benzyl alcohol (1%) under magnetic agitation overnight. Additionally, a dispersion of formulation F7 containing 30-mg/mL NTX was prepared using the same medium.

#### 2.6.2. Preparation of Porcine Ear Skin

**Figure 1:**
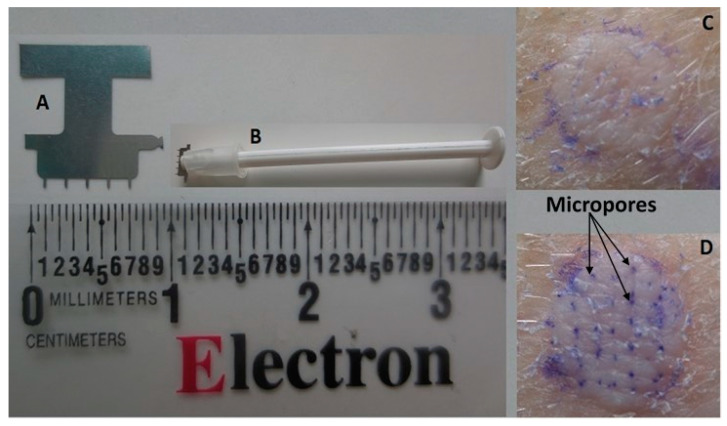
(**A**) In-line microneedle (MN) array containing 5 MNs of µm length, (**B**) MN array with applicator made in-house, (**C**) intact skin stained with gentian violet, and (**D**) MN-treated skin stained with gentian violet, showing micropore formation.

Excised porcine ear skin for all in vitro permeation studies was obtained from a local slaughterhouse and stored at −80 °C until use. Skin samples were thawed on the day of use and were dermatomed (TCM 3000BL, Nouvag AG, Goldach, Switzerland) to a thickness of 1-mm skin before use. For MN-treated skin, an “in-plane” array of 5 stainless steel MNs, 750-µm length, was used (Tech-Etch Inc, Plymouth, MA, USA). The array was applied a total of 20 times to create 100 microchannels on a skin area of 0.64 cm^2^ (corresponding to the diffusion area of the in-line diffusion cells). To create the microchannels, the array was first applied 10 times in one direction, the skin was turned approximately 45°, and then, an additional 10 MN applications were performed. To verify micropore formation, additional skin samples were treated with MNs and then stained with gentian violet dye to allow visualization of the micropores. The dye was allowed to stand on the skin surface for 1 min for adequate staining, and the skin surface was then cleaned with isopropyl alcohol swabs to remove excess dye. Skin samples that were not treated with MNs were stained with the same technique for comparison. An image of a sample MN array and MN-treated skin is shown in Figure 1.

#### 2.6.3. In Vitro Skin Permeation

In order to evaluate the influence of the chitosan polymer concentration on NTX flux through the microchannels created from MN insertion, MN-treated porcine skin was mounted into the in-line diffusion cells with the stratum corneum side facing upwards. Five-hundred microliters of dispersions of formulations F7, F19, and F27 (each containing 12-mg/mL NTX) were applied to the MN-treated skin area. Samples were collected for 48 h and NTX content in the receiver medium was quantified via HPLC. Cumulative NTX vs. time was plotted, and flux was determined from the steady-state portion of the curve. Based on results from this experiment, an additional skin permeation study was performed using a higher concentration of NTX; specifically, a dispersion of formulation F7 containing 30-mg/mL NTX. All other conditions for the permeation studies were the same as those for the in vitro release studies, and intact skin served as the control for this study. All experiments were performed in triplicate.

#### 2.6.4. NTX Recovery from Porcine Skin

NTX concentration in the skin was quantified upon completion of the in vitro skin permeation studies. The diffusion area was isolated, blotted dry, and then tape-stripped twice to remove residual formulation from the skin surface (Scotch ½” Magic Tape, 3M, Maplewood, MN, USA). The skin was weighed, cut into small pieces, and extracted in methanol overnight under agitation. Each sample was further sonicated for 20 min, centrifuged (AccuSpin Micro 17R, Fisher Scientific, Hampton, NH, USA), appropriately diluted, and syringe-filtered. NTX content was quantified using HPLC. Experiments were performed in triplicate.

### 2.7. HPLC Method

NTX was quantified using HPLC analysis on a Shimadzu LC-2030 Prominence iSeries system (Shimadzu, Kyoto, Japan) equipped with a LC-2030 pump, autosampler, and UV detector; data analysis was performed using Shimadzu^®^ LC Solution software (Shimadzu, Kyoto, Japan, Ver. 5.92). Reverse phase chromatographic separation was carried out using a Kinetex^®^ C18 100 Å column (150 × 4.6 mm i.d., 5μm; Phenomenex, Torrance, CA, USA). The mobile phase (A: 0.1% TFA and 0.06% *v*/*v* TEA in water and B: acetonitrile) was used in a gradient elution as follows: phase B concentration increased from 10% to 30% over 4 min, maintained at 30% for 1 min, and then decreased to 10% until 6 min for equilibration. Flow rate was 1.0 mL/min, oven temperature maintained at 30 °C, and injection volume was 10 µL. NTX was detected at wavelength of 226 nm with a retention time of 3.62 min. The standard curve was linear across the range of 0.4 to 100 µg/mL (R^2^ ≥ 0.99). Method accuracy (relative error (RE), %) was <1.5%, and within-day and between-day precision (relative standard deviation (RSD), %) was <2.5%.

### 2.8. Statistical Analysis

Results obtained were subjected to ANOVA and Student’s *t*-test analysis, and parameter estimation was done with general linear modeling using GraphPad Prism 8 (GraphPad Software, San Diego, CA, USA) and Minitab^®^ 18 software (Minitab Inc., State College, PA, USA), respectively. *p* < 0.05 was considered statistically significant.

## 3. Results and Discussion

### 3.1. Quality Attributes and Characterization of Chitosan-NTX Microspheres

#### 3.1.1. Summary of Quality Attributes

A summary of chitosan-NTX microsphere quality attributes and DoE statistical analysis results are presented in Table 1 and Table 3, respectively. Model equations generated after statistical analysis for each quality attribute are provided in the Supplementary Materials. In general, for different combinations in the experimental design, spray-dried microsphere yield ranged from 17% to 43%, mean hydrodynamic diameter by dynamic light scattering ranged from 1.7 to 9.2 µm, and polydispersity index was >0.4. Mean zeta potential ranged from +34 to +66 mV, while mean outlet air temperature ranged from 60 to 98 °C. Mean encapsulation efficiency was between 70% and 87%, mean drug-loading capacity ranged from 10% to 43%, and mean percent cumulative in vitro NTX release over 24 h ranged from 63% to 94%.

#### 3.1.2. Influence of Parameters on Microspheres Quality Attributes

**Figure 2:**
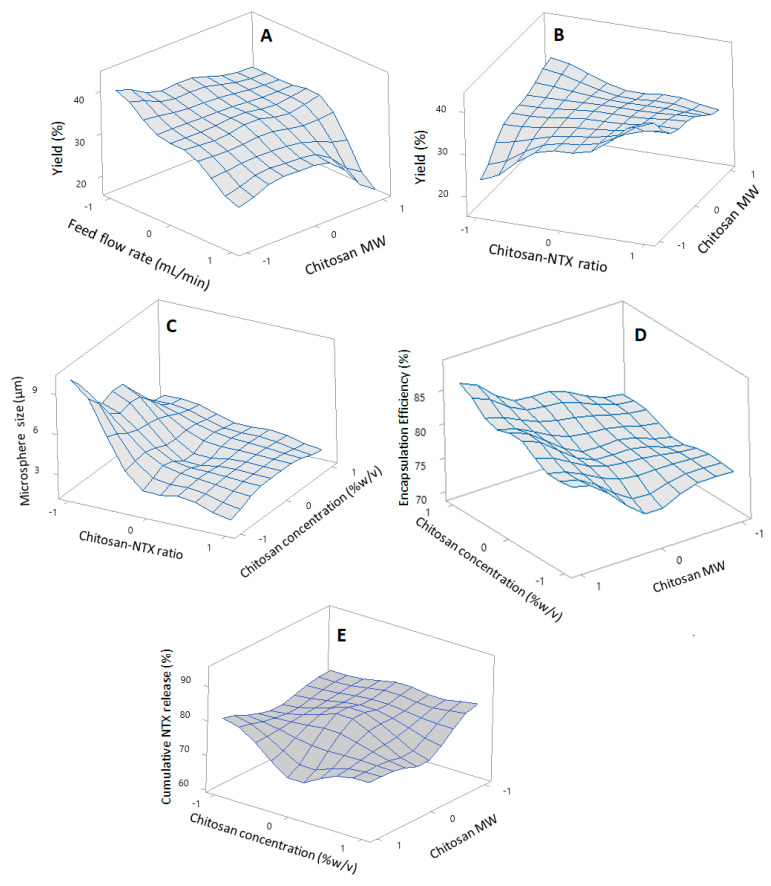
Response surface plots of (**A**) yield vs. chitosan MW and feed flow rate, (**B**) yield vs. chitosan MW and chitosan-NTX ratio, (**C**) microsphere size vs. chitosan concentration and chitosan-NTX ratio, (**D**) encapsulation efficiency vs. chitosan MW and concentration, and (**E**) % cumulative in vitro NTX release vs. chitosan MW and concentration. MW = molecular weight and NTX = naltrexone.

The influence of selected variables on the quality attributes of chitosan-NTX microspheres were examined in this study. Benchtop spray-dryers such as the Buchi spray-dryer used in this study generally produce a powder yield in the range of 8% to 84% [23,28,29]. In this study, feed flow rate significantly influenced the powder yield (*p* = 0.003). Increasing the feed flow rate from 4 to 8 mL/min decreased the powder yield (Figure 2A), similar to previous reports [30,31]. This may be due to the fact that at higher feed flow rates there is an increase in feed load, causing slower heat and mass transfer, leading to insufficient drying and sticking to chamber walls that consequently lowers the yield [32,33,34]. Moreover, the microsphere yield was influenced by an interaction between chitosan MW and ratio of chitosan:NTX (Figure 2B). Higher yields were achieved with a decrease from high to low-MW chitosan and increase from a low to high level of chitosan:NTX (an increase in chitosan proportion in the dry weight of the chitosan-NTX mix). A study from Desai’s group reported similar results, in which a higher chitosan microsphere yield was achieved using low MW chitosan vs. medium and high MW chitosan in a spray-drying process [35]. While this phenomenon may be difficult to explain, it could be related to the bulk density/lightness of the chitosan polymer at different MW. Aranaz et al. demonstrated that larger amounts of the lightest chitosan polymer were lost and not trapped by the spray-dryer aspirator, leading to a lower product yield [36]. Low-MW chitosan appears to be less bulky and heavier than medium and high-MW chitosan; likewise, NTX appears to be lighter and less bulky than the chitosan polymers. Thus, a combination of these two situations could have led to the product yield observed—a decrease in chitosan MW from high to low might have led to the production of heavier microspheres that are easily trapped by the spray-dryer aspirator, while higher proportions of “heavier” chitosan polymers in comparison to NTX might have produced the same effect, a combination effect leading to a higher product yield. The equation describing the influence of the tested parameters on the powder yield is:

Yield (%) = 32.672 − 1.09 A + 1.31 B + 1.96 C − 5.27 D + 3.92 A × B − 6.44 A × C − 0.32 A×D − 1.24 B × C + 0.51 B × D + 2.37 C × D(4)

Outlet air temperature was significantly influenced by the feed flow rate (*p* < 0.0001). Outlet air temperature has an important effect on the moisture content of spray-dried powders, and it is important to have an optimal outlet air temperature in a spray-drying process. A decrease in outlet air temperature often leads to an increased powder moisture content [37]. The current study demonstrated that an increase in the feed flow rate from 4 to 8 mL/min led to a significant decrease in the outlet air temperature, which is consistent with other studies [38]. This may also explain the low product yield observed at higher feed flow rates. Increased powder moisture associated with a very low outlet air temperature may cause powder to stick to the chamber walls [31,39,40]. The equation describing the influence of the tested parameters on the outlet air temperature is:

Outlet air temperature (°C) = 77.11 + 0.25 A + 0.75 B + 2.42 C − 13.42 D + 0.25 A × B − 3.25 A × C − 0.25 A × D − 3.25 B × C − 0.25 B × D − 2.75 C × D(5)

The chitosan:NTX ratio significantly influenced the microsphere size (*p* = 0.001). A decrease from the high-level chitosan:NTX ratio (+1, five-parts chitosan to one-part NTX) to a low-level (−1, one-part chitosan to one-part NTX) led to an increased microsphere size (Figure 2C). This could be related to the feed concentration of the chitosan-NTX dispersion. High feed concentrations can cause an increase in viscosity, which can consequently lead to the formation of larger droplet sizes and the production of powders with larger sizes during spray-drying [20,41,42]. This was observed in several formulations in the current study. To explain this, formulations F13, F1, and F10 had 0.5% *w*/*v* of medium MW chitosan at chitosan:NTX ratios of 5, 3, and 1 and DoE levels of +1, 0, and −1, respectively. This corresponded to actual feed concentrations of 0.58%, 0.63%, and 1.0% *w*/*v* and microsphere sizes of 2.08 ± 0.15, 2.69 ± 0.35, and 9.20 ± 1.33 µm, respectively. The microspheres were polydispersed, which is consistent with other studies of spray-dried chitosan [36,43]. The equation describing the influence of tested parameters on the microsphere size is:

Microsphere size (µm) = 3.626 − 0.184 A − 0.460 B − 2.117 C − 0.215 D − 0.442 A × B + 0.617 A × C − 0.131 A × D + 1.605 B × C − 0.039 B × D + 1.345 C × D(6)

Zeta potential was not significantly influenced by any of the variables evaluated and was positive for all formulations; this is typical of chitosan polymers [35]. The equation describing the influence of the tested parameters on the zeta potential is:

Zeta potential (mV) = 51.06 + 0.13 A + 3.96 B + 6.92 C + 2.10 D − 9.75 A × B + 2.95 A × C − 4.65 A × D + 2.55 B × C − 5.47 B × D + 2.08 C × D(7)

Chitosan MW and concentration individually influenced the encapsulation efficiency (*p* = 0.009 and 0.042, respectively). Increased chitosan MW and concentration led to an increase in the NTX encapsulation efficiency (Figure 2D), which is consistent with other studies [20,44]. The influence of the MW could be due to the relationship between the MW and viscosity of chitosan. Higher MW chitosan tends to be more viscous than lower MW chitosan, with an increase in viscosity potentially leading to more efficient NTX encapsulation [35]. Similarly, an increased chitosan concentration may lead to an increase in viscosity and availability of more polymer to entrap NTX [20,35]. Thus, a combination effect of increased viscosity caused by increased chitosan MW and concentration could have led to the increase in the encapsulation efficiency observed. Although the initial results showed that a higher product yield may be achieved using low-chitosan MW (which may lead a to lower encapsulation efficiency), it is important that the totality of variables that may influence the microsphere quality attributes be carefully considered to choose the right formulation that fits and produces the optimum results for our intended drug delivery purpose. Thus, the in vitro release and skin permeation of these formulations were also considered to choose an ideal formulation in this study. The equation describing the influence of the tested parameters on the encapsulation efficiency is:

Encapsulation Efficiency (%) = 77.959 + 4.12 A + 3.04 B − 1.37 C − 1.35 D + 0.98 A × B + 0.28 A × C − 2.55 A × D − 0.72 B × C + 2.46 B × D + 0.82 C × D(8)

Drug-loading capacity was significantly influenced by the chitosan:NTX ratio (*p* < 0.0001), with a decrease from a high (+1, five-parts chitosan to one-part NTX) to low (−1, one-part chitosan to one-part NTX) chitosan-NTX ratio, leading to a higher drug-loading capacity. No relationship was observed between the encapsulation efficiency and drug-loading capacity, further showing that these quality attributes were influenced by the process and formulation variables used in this study. The equation describing the effect of the tested parameters on the drug-loading capacity is:

Drug-loading capacity (%) = 19.89 + 1.02 A + 0.90 B − 14.29 C − 0.34 D + 0.20 A × B − 0.87 A × C − 0.51 A × D − 1.39 B × C + 0.49 B × D + 0.35 C × D(9)

Percent cumulative in vitro NTX release at pH 7.4 for 24 h in SnakeSkin^®^ cellulose dialysis membrane (10,000 Da MWCO, Thermo Scientific, USA) shows that the chitosan MW significantly (*p* = 0.043) influenced the NTX release, as a higher percent NTX release was achieved with a change from a high to low MW chitosan (Figure 2E). This result is in agreement with other studies that demonstrated a slower drug release from high MW chitosan and could be due to a high-density polymer shell around the drug particles, which may retard the fluid ingress into the polymer shell and, thus, decrease the drug release [20,45]. The equation describing the effect of the tested parameters on the in vitro cumulative NTX release is:

Cumulative NTX release (%) = 76.97 − 4.65 A − 1.36 B − 3.31 C + 1.94 D − 2.24 A × B + 2.47 A × C − 1.85 A × D − 4.48 B × C − 0.48 B × D − 3.91 C × D(10)

Other studies were performed to understand the necessary parameters of the formulations to choose an ideal formulation. An ideal formulation would be one that has a high NTX encapsulation efficiency and drug-loading capacity, optimum/high NTX in vitro release and skin flux, and can be easily manipulated for dosing/application.

### 3.2. Shape and Surface Morphology

**Figure 3:**
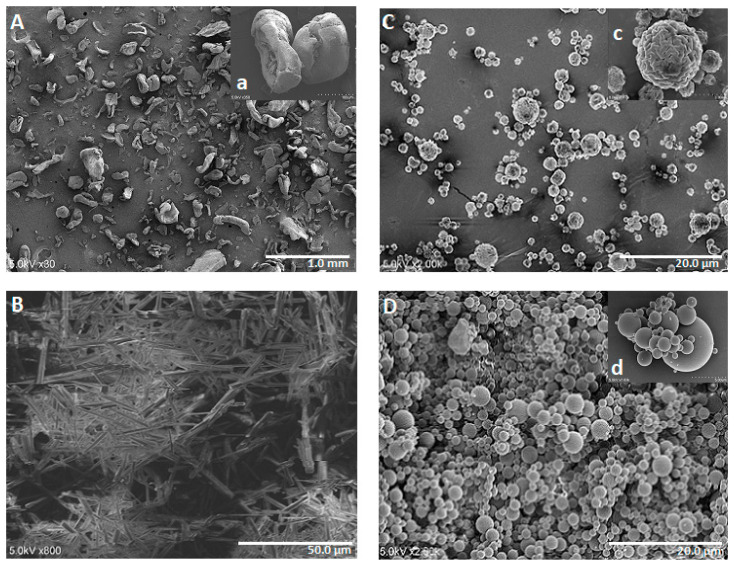
SEM photomicrographs of materials used in the studies: (**A**) pure high MW chitosan, (**B**) NTX hydrochloride powder, (**C**) spray-dried blank high MW chitosan, and (**D**) spray-dried microparticles of Formulation F7.

Figure 3 shows the SEM photomicrographs of pure high MW chitosan, NTX powder, blank spray-dried chitosan, and spray-dried chitosan-NTX microspheres (Formulation F7). Figure 3A shows pure high MW chitosan with irregularly shaped and rough-surfaced particles of diverse sizes, while Figure 3B shows long, crystal-like structures of pure NTX. SEM photomicrographs of blank spray-dried chitosan microspheres (Figure 3C) show spherical and wrinkled surfaces appearing in the form of troughs and ridges. This type of surface morphology exhibited by blank spray-dried chitosan microspheres is in-line with previous reports [35,43,46].

SEM photomicrographs of spray-dried chitosan-NTX microspheres (Formulation F7, Figure 3D) show a polydisperse particulate system with diameters ranging from 0.35 to 8.56 µm (mean diameter of 2.63 ± 1.55 µm), further confirming the polydisperse results obtained from the dynamic light-scattering characterization.

The microspheres, however, have particles with regular spherical shapes and smooth surfaces, showing the effectiveness of the spray-drying process in converting irregularly shaped chitosan and crystal-like NTX into regularly shaped microspheres. There were no free crystal-like NTX (Figure 3D) present on the chitosan-NTX microsphere surfaces, which infers an amorphous nature of the microspheres, as powders produced from spray-drying are generally known to be amorphous in nature [20,47]. Formulations F19 and F27 had regular spherical-shaped particles with similar smooth surfaces as those observed with Formulation F7. Photomicrographs of Formulations F19 and F27 are included in the Supplementary Materials.

### 3.3. Solid State Characterization

#### 3.3.1. FT-IR Spectra

**Figure 4:**
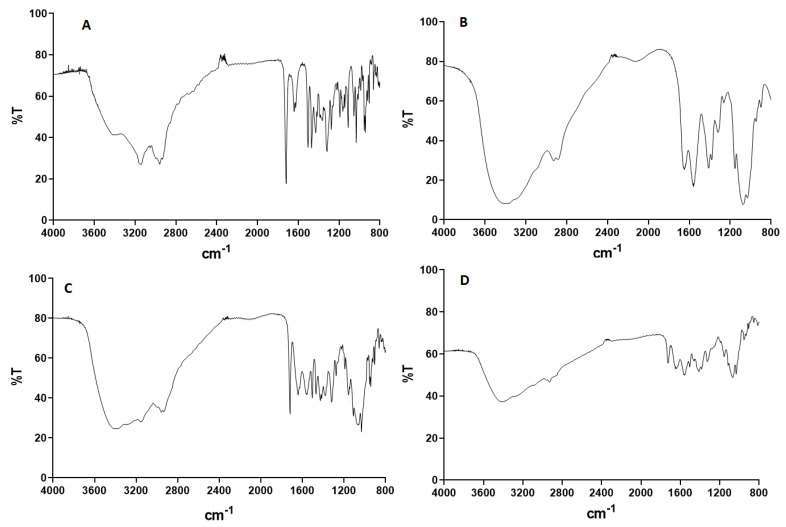
FT-IR spectrum of (**A**) NTX HCl, (**B**) spray-dried chitosan (high MW), (**C**) physical mixture of NTX HCl/spray-dried chitosan (high MW) (1:1), and (**D**) spray-dried chitosan-NTX microsphere (Formulation F7). NTX = naltrexone and MW = molecular weight.

Figure 4 shows the FT-IR spectra of NTX powder, blank spray-dried high MW chitosan, a physical mixture of NTX and blank spray-dried chitosan (1:1), and spray-dried chitosan-NTX microspheres (Formulation F7). Figure 4A shows the characteristic peaks typical of NTX near wavenumbers 1717, 1660, 1504, 1314, and 1274 cm^−1^ [48]. The high intensity peak at 1717 cm^−1^ is due to the stretching absorption of C=O of the saturated cyclic aliphatic ketone, while the peak at 1660 cm^−1^ shows a C=C stretching band of C=C-O-H. There is a C=C stretching of the aromatic ring that appeared at peak 1504 cm^−1^, an O-H bending absorption appearing at 1314 cm^−1^, and a C-O-C asymmetric stretching peak observed at 1274 cm^−1^ [48].

Figure 4B shows the spectrum of blank spray-dried high MW chitosan with a strong broad band at ~3428 cm^−1^; two bands at about wave numbers of 2921 and 2874 cm^−1^; and other bands at 1652, 1597, 1420, 1377, 1322, 1154, and 1094 cm^−1^, respectively. The strong band at 3428 cm^−1^ corresponds to a stretching vibration of -OH, an extension vibration of N-H and the intermolecular hydrogen bonds of the polysaccharides. The bands at about 2921 and 2874 cm^−1^ correspond to an asymmetric and symmetric C-H stretching vibrations. There was a characteristic -NH-bending vibration of the primary amine groups at about 1652 and 1597 cm^−1^, while the bands at 1420, 1377, and 1322 cm^−1^ corresponded to the C-N stretching vibrations. A C-O stretching vibration in the secondary alcohol was observed at about 1154 cm^−1^, while a C-O starching vibration in the alcohol was also seen at about 1094 cm^−1^ [49].

Figure 4C shows the FT-IR spectrum of the physical mixture of NTX powder and blank spray-dried chitosan (high MW) with superimposable characteristic absorption bands of individual pure NTX powder (Figure 4A) and blank spray-dried chitosan (Figure 4B), implying no interaction between the drug and polymer. Figure 4D shows the FT-IR spectrum of spray-dried chitosan-NTX microspheres with characteristic bands observed in pure NTX and blank spray-dried chitosan but at low intensities. While the intensities of the bands were reduced, there were no new bands observed in the spectrum, confirming that no new chemical bonds were formed between NTX and chitosan after spray-drying.

#### 3.3.2. DSC Thermograms

**Figure 5:**
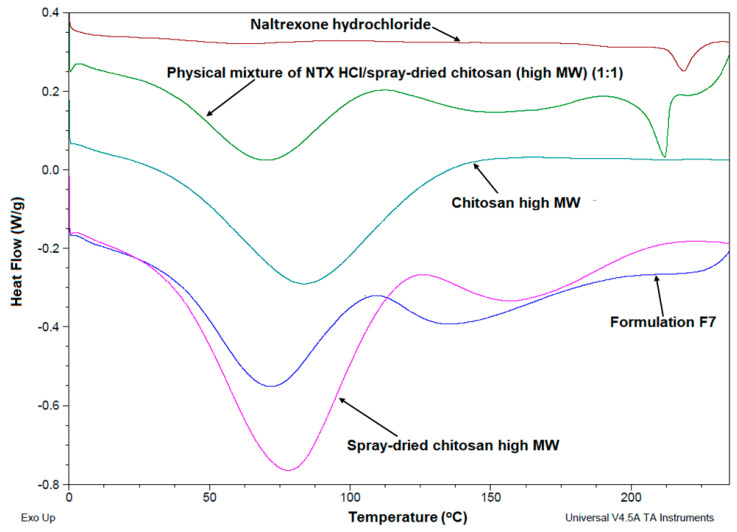
Differential scanning calorimetry (DSC) thermograms of NTX HCl, spray-dried chitosan (high MW), a physical mixture of NTX HCl/spray-dried chitosan (high MW) (1:1), and spray-dried chitosan-NTX (Formulation F7). NTX = naltrexone and MW = molecular weight.

Thermal properties of pure NTX, pure high MW chitosan, blank spray-dried chitosan, and spray-dried chitosan-NTX microspheres were studied using DSC from 0 to 240 °C. Figure 5 shows the DSC thermograms of NTX powder, pure high MW chitosan and blank spray-dried chitosan, a physical mixture of NTX and blank spray-dried chitosan (1:1), and spray-dried chitosan-NTX microspheres (Formulation F7).

The NTX thermogram showed a sharp endothermic peak at ~219 °C, indicating the melting of NTX. Pure chitosan showed a broad endothermic peak only at ~75 °C, while spray-dried chitosan showed two peaks at 75 and 160 °C. The endothermic peak at 75 °C can be ascribed to water loss from the chitosan polymer during the heating process, as the chitosan polysaccharide has a disordered structure with a strong affinity for residual water [50]. The second endothermic peak at 160 °C may be due to a slight glass transition of chitosan (T_g_); however, affirming the glass transition temperature of the chitosan polymer is quite cumbersome, because as a natural polymer, properties such as the MW, crystallinity, and deacetylation degree often result in a wide range of variations in chitosan T_g_ [51]. In fact, this slight glass transition was not observed in pure chitosan that was not spray-dried. The physical mixture of NTX and blank spray-dried chitosan (1:1) showed three endothermic peaks at ~75, 160, and 213 °C, corresponding to a loss of water from the chitosan polymer, slight T_g_, and melting of free NTX in the mixture. However, spray-dried chitosan-NTX microspheres (Formulation F7) showed only two endothermic peaks at 75 and 160 °C, corresponding to the initial water loss from the chitosan polymer and a slight T_g_. There was, however, no peak corresponding to the melting of NTX within the range of temperatures studied (0–240 °C), which indicates an amorphous dispersion of NTX within the chitosan microspheres. This may be due to the fact that powders produced from spray-drying are generally known to be amorphous in nature [20,47].

### 3.4. In Vitro Skin Permeation

In order to evaluate the influence of chitosan MW on in vitro NTX flux from chitosan-NTX microspheres applied to MN-treated porcine skin, formulations F7, F19, and F27 were chosen and initially evaluated at an NTX concentration of 12 mg/mL. These formulations were chosen for the following reasons: they were prepared using high (F7), medium (F19), and low MW (F27) chitosan but at the same polymer concentration (0.7 %*w*/*v*) and chitosan-NTX ratio (1:1). These formulations also had high encapsulation efficiencies, in addition to similarly high drug-loading capacities. Formulations F7 and F27 had comparable characteristics in terms of mean diameters (both larger than Formulation F19), though it is important to note that, in quality-by-design, the totality of tested variables and their effects on the formulation responses within the design of the experiment is highly important rather than looking at the effects on a response in a single formulation within the design to make formulation selection decisions. Table 4 shows the attributes of Formulations F7, F19, and F27.

In vitro NTX skin flux from Formulations F7, F19, and F27 containing 12-mg/mL NTX applied to MN-treated porcine ear skin were 3.89 ± 0.96, 4.24 ± 1.57, and 3.28 ± 0.98 µg/cm^2^·h, respectively. There was no significant difference in NTX flux between these three formulations when applied to MN-treated skin, despite that in vitro NTX release from microspheres containing 2-mg/mL NTX showed that increased chitosan MW led to a decreased cumulative NTX release over 24 h. This result may be explained by the properties of the hydrophilic natural polymers such as chitosan, which swell rapidly in aqueous mediums, forming a gel-like structure that can control and slow down drug release [14]. The higher amount of chitosan (12 mg/mL) in the formulations used in this skin permeation study vs. that of in vitro release studies (2 mg/mL) may have canceled out the effect that chitosan MW had on the NTX flux. To support this hypothesis, no significant difference in percent cumulative the in vitro NTX release in the SnakeSkin^®^ cellulose dialysis membrane was observed from Formulations F7, F19, and F27 containing 12-mg/mL NTX in 24 h. Based on these results, Formulation F7 prepared using high MW chitosan was chosen as the ideal formulation for further study. Chitosan MW as a categorical variable led to the selection of Formulations F7 (high MW), F19 (medium MW), and F27 (low MW), because these possessed the highest encapsulation efficiencies and drug-loading contents. While Formulation F7 had a lower NTX in vitro release at lower NTX concentrations, no significant difference was observed in the NTX in vitro release and the skin flux at higher NTX concentrations between Formulations F7, F19, and F27. In addition, F7 was easier to manipulate in preparing a formulation containing higher NTX concentrations, making F7 a formulation that fit our previously defined criteria.

In order to facilitate a higher NTX flux, skin permeation studies were performed using a dispersion of Formulation F7 containing 30-mg/mL NTX. The flux of NTX from Formulation F7 containing 30-mg/mL NTX applied to MN-treated skin was 11.57 ± 2.24 µg/cm^2^·h, while no NTX was detected in the receiver medium of the intact skin treated with Formulation F7. MN treatment of the porcine ear skin thus facilitated a significant flux enhancement from Formulation F7. This result is expected, as MN treatment facilitates the creation of microchannel pathways through the stratum corneum that allow the effective diffusion of NTX across the skin [6,7]. The lag time of NTX from Formulation F7 was 1.52 ± 0.35 h, demonstrating that F7 will begin to have a clinical effect in less than 2 h of application.

A study published by Wermeling et al. reported an in vitro NTX steady-state flux of 14.7 ± 4.9 µg/cm^2^·h in MN-treated skin using a 16% *w*/*v* (160 mg/mL) NTX gel prepared with an aqueous medium containing 60.75% *v*/*v* propylene glycol (a skin permeation enhancer) and 1% *w*/*w* benzyl alcohol [4]. In our current study, we prepared chitosan-NTX microspheres containing 3% *w*/*v* (30 mg/mL) NTX in an aqueous medium containing 20% *v*/*v* polyethylene glycol and 1% *v*/*v* benzyl alcohol. We report a NTX steady-state flux of 11.57 ± 2.24 µg/cm^2^·h in MN-treated skin, which is not notably different from the NTX flux reported in the Wermeling et al. study, considering the significantly higher NTX concentrations in the gel used in their study. Thus, we achieved a nearly comparable NTX steady-state flux in vitro using a significantly lower NTX concentration.

**Figure 6:**
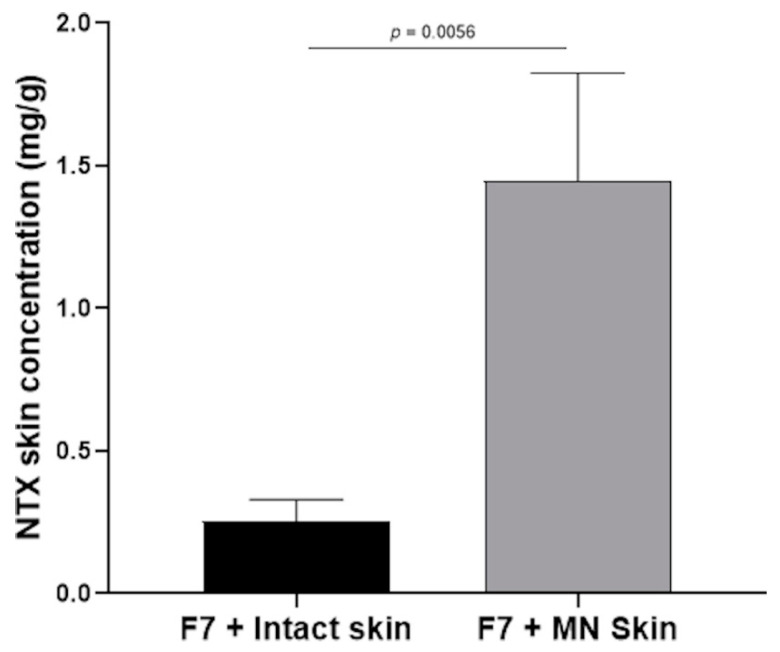
NTX recovery from intact and MN-treated skin following the application of Formulation F7 containing 30-mg/mL NTX (*n* = 3, data represented as mean ± SD). NTX = naltrexone and MN = microneedle.

The concentrations of NTX extracted from intact and MN-treated skin are shown in Figure 6. Following the application of 500 µL of Formulation F7 (30-mg/mL NTX), NTX recovered from skin was 0.25 ± 0.08 and 1.45 ± 0.38 mg/g of porcine skin for intact and MN-treated skin, respectively. NTX recovered from the skin showed similar trends as the NTX flux. NTX recovered from MN-treated skin with Formulation F7 were significantly higher than from intact skin (*p* = 0.0056). NTX recovered from the skin represent a skin reservoir that can contribute to a continuous NTX release across the skin, which can help to maintain the delivery of therapeutic concentrations of NTX.

## 4. Conclusions

This study demonstrated the process for obtaining chitosan-NTX microspheres using a spray-drying technique. Spray-drying produced spherical and smooth-surfaced chitosan-NTX microspheres with amorphous characteristics. The study shows that input variables (chitosan MW, chitosan concentration, chitosan-NTX ratio, and feed flow rate) individually or in combination influenced the yield, size, encapsulation efficiency, drug-loading capacity, and cumulative in vitro NTX release from the microspheres. Powder yield, size, and encapsulation efficiency were mainly affected by the feed flow rate, feed concentration, and chitosan MW and concentration, respectively. MN treatment of the skin significantly increased the NTX flux from chitosan-NTX microspheres in comparison to the intact skin. The application of MNs in combination with NTX formulations such as the type described in this study could improve patient compliance by preventing the drawbacks of oral and intramuscular NTX and support a sustained delivery of NTX across the skin.

## Figures and Tables

**Table 1:** Responses for randomized 27-experiment Box Behnken design of chitosan-naltrexone (NTX) microspheres. *n* = 3 and data represented as mean ± SD. EE = encapsulation efficiency.

Run	A	B	C	D	Yield (%)	Mean Diameter (µm)	Mean zeta Potential (mV)	EE (%)	Drug-Loading Capacity (%)	Outlet Air Temperature (°C)	NTX Release (%)	Dry Batch Weight (g)
F1	0	−1	0	−1	38.6	2.69 ± 0.35	52.95 ± 2.62	78.0 ± 0.2	15.6 ± 0.04	84	69.4 ± 4.4	1.5
F2	0	0	0	0	23.4	2.03 ± 0.39	59.93 ± 6.05	84.2 ± 0.3	16.8 ± 0.07	73	74.7 ± 1.1	1.5
F3	1	0	0	−1	33.2	1.73 ± 0.13	55.33 ± 1.80	86.5 ± 0.5	17.3 ± 0.10	87	65.1 ± 3.9	1.5
F4	1	1	0	0	39.0	2.25 ± 0.15	60.37 ± 6.93	86.1 ± 4.5	17.2 ± 0.91	80	74.9 ± 20.6	2
F5	0	0	−1	−1	33.7	8.35 ± 0.41	42.03 ± 5.22	80.1 ± 1.1	40.0 ± 0.55	88	81.5 ± 7.1	1.4
F6	1	0	1	0	29.4	3.34 ± 0.19	47.45 ± 7.14	88.3 ± 3.3	12.6 ± 0.47	76	65.8 ± 4.0	1.4
F7	1	0	−1	0	35.4	7.31 ± 0.89	34.23 ± 1.35	85.1 ± 1.4	42.5 ± 0.72	80	72.1 ± 4.2	2
F8	0	−1	0	1	29.7	2.84 ± 0.10	59.70 ± 6.30	73.6 ± 0.6	14.7 ± 0.11	60	67.2 ± 0.6	1.5
F9	0	0	0	0	33.1	2.71 ± 0.23	60.75 ± 0.21	73.8 ± 2.6	14.8 ± 0.52	85	81.4 ± 0.8	1.5
F10	0	−1	−1	0	31.6	9.20 ± 1.33	43.67 ± 0.46	71.0 ± 0.0	35.5 ± 0.00	75	79.7 ± 3.4	1.4
F11	0	0	1	1	30.0	3.21 ± 0.22	61.67 ± 5.23	70.5 ± 0.3	10.1 ± 0.05	60	79.8 ± 1.1	1.4
F12	0	0	1	−1	33.6	2.93 ± 0.18	59.47 ± 1.68	69.8 ± 1.7	10.0 ± 0.24	98	82.9 ± 6.2	1.4
F13	0	−1	1	0	34.5	2.08 ± 0.15	44.40 ± 5.26	70.2 ± 3.2	10.1 ± 0.46	89	86.5 ± 3.1	1.87
F14	−1	0	0	1	25.4	3.84 ± 0.20	61.33 ± 0.91	72.3 ± 1.9	14.5 ± 0.39	60	88.6 ± 6.6	1.5
F15	1	0	0	1	17.0	2.48 ± 0.15	57.05 ± 2.47	73.7 ± 0.1	14.7 ± 0.02	63	71.5 ± 1.4	1.5
F16	−1	0	0	−1	40.3	2.64 ± 0.16	66.50 ± 0.36	74.9 ± 3.5	15.0 ± 0.70	83	74.8 ± 6.1	1.5
F17	1	−1	0	0	32.2	3.23 ± 0.11	63.20 ± 2.17	80.9 ± 2.8	16.2 ± 0.55	75	80.7 ± 3.2	1.5
F18	0	1	−1	0	41.0	3.67 ± 0.23	56.23 ± 7.57	85.1 ± 0.3	42.6 ± 0.17	80	74.0 ± 4.8	1.4
F19	0	0	−1	1	20.7	3.67 ± 0.13	46.90 ± 2.51	77.5 ± 1.9	38.8 ± 0.95	61	94.0 ± 4.2	1.6
F20	−1	−1	0	0	37.9	2.19 ± 0.33	57.10 ± 1.41	75.5 ± 1.9	15.1 ± 0.38	81	78.8 ± 13.2	1.5
F21	0	0	0	0	35.6	2.98 ± 0.08	61.30 ± 2.97	81.2 ± 1.7	16.2 ± 0.33	82	76.2 ± 5.6	2
F22	0	1	0	1	32.7	2.71 ± 0.21	57.40 ± 7.38	80.9 ± 3.2	16.2 ± 0.64	61	74.0 ± 5.0	1.5
F23	−1	1	0	0	29.0	2.92 ± 0.24	63.03 ± 5.24	76.7 ± 4.7	15.3 ± 0.94	85	82.0 ± 9.3	2
F24	0	1	0	−1	39.5	3.14 ± 0.09	63.40 ± 0.71	75.5 ± 3.9	15.1 ± 0.79	86	78.2 ± 2.0	1.5
F25	−1	0	1	0	43.2	2.20 ± 0.21	57.73 ± 2.50	77.0 ± 1.6	11.0 ± 0.22	79	72.7 ± 6.3	1.4
F26	0	1	1	0	38.9	2.52 ± 0.27	56.47 ± 4.25	81.5 ± 4.3	11.6 ± 0.61	81	62.9 ± 11.8	1.4
F27	−1	0	−1	0	23.4	8.29 ± 0.70	52.17 ± 0.21	74.9 ± 4.8	37.5 ± 2.42	70	89.0 ± 1.5	2

**Table 2:** Description of variables and levels. L, M, and H depicts low, medium, and high molecular weight chitosan.

Coded Variables	Uncoded Variables	−1	0	1
A	Chitosan molecular weight	L	M	H
B	Chitosan concentration (%*w*/*v*)	0.5	0.7	0.9
C	Chitosan-NTX ratio	1	3	5
D	Feed flow rate (mL/min)	4	6	8

**Table 3:** Individual influence of parameters on chitosan-NTX microsphere properties. *n* = 3 and data represented as mean ± SD.

Individual Effects on							
Input Variables	Yield (%)	Diameter (µm)	Zeta Potential (mV)	Outlet Air Temperature (°C)	EE (%)	Drug-Loading Capacity (%)	NTX Release (%)
Chitosan MW	*p* = 0.476	*p* = 0.718	*p* = 0.975	*p* = 0.877	*p* = 0.009 *	*p* = 0.579	*p* = 0.043 *
Chitosan concentration	*p* = 0.394	*p* = 0.372	*p* = 0.352	*p* = 0.643	*p* = 0.042 *	*p* = 0.626	*p* = 0.532
Chitosan-NTX ratio	*p* = 0.207	*p* = 0.001 *	*p* = 0.113	*p* = 0.147	*p* = 0.335	*p* < 0.0001*	*p* = 0.138
Feed flow rate	*p* = 0.003 *	*p* = 0.673	*p* = 0.618	*p* < 0.001 *	*p* = 0.341	*p* = 0.854	*p* = 0.373

* *p*-values < 0.05 are statistically significant. MW = molecular weight.

**Table 4:** Characteristics of selected formulations. *n* = 3 and data displayed as mean ± SD.

Formulations	Chitosan MW (kDa)	Yield (%)	Mean Diameter (µm)	Encapsulation Efficiency (%)	Drug-Loading Capacity (%)	Cumulative in Vitro NTX Release (%)	* NTX Flux in MN-Treated Porcine Skin (µg/cm^2^·h)
F7	310–375	35.4	7.31 ± 0.89	85.1 ± 1.4	42.5 ± 0.72	72.1 ± 4.2	3.89 ± 0.96
F19	190–310	20.7	3.67 ± 0.13	77.5 ± 1.9	38.8 ± 0.95	94.0 ± 4.2	4.24 ± 1.57
F27	50–190	23.4	8.29 ± 0.70	74.9 ± 4.8	37.5 ± 2.42	89.0 ± 1.5	3.28 ± 0.98

* Skin samples were treated with microspheres containing 12-mg/mL NTX. NTX = naltrexone and MN = microneedle.

## Supplementary Materials

The following are available online at https://www.mdpi.com/1999-4923/12/6/496/s1: Figure S1: SEM photomicrographs of formulation (A) F19 and (B) F27.
