# Supplementary material for: Design and Characterization of Spray-Dried Chitosan-Naltrexone Microspheres for Microneedle-Assisted Transdermal Delivery
Source: Pharmaceutics. 2020 May 29;12(6):496. doi: 10.3390/pharmaceutics12060496 (PMC7355536; doi:10.3390/pharmaceutics12060496)
Supplement: Supplementary file 1 [file pharmaceutics-12-00496-s001.pdf]

pharmaceutics

Supplementary Materials: Design and Characterization of Spray-Dried Chitosan-Naltrexone Microspheres for Microneedle-Assisted Transdermal Delivery

Abayomi T. Ogunjimi, Jennifer Fiegel and Nicole K. Brogden *

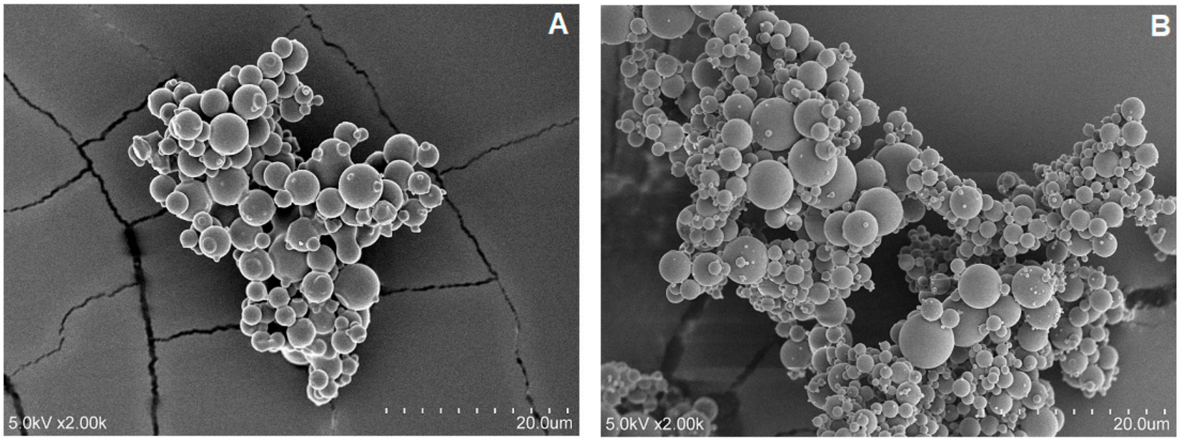

Figure S1. SEM photomicrographs of formulation (**A**) F19 and (**B**) F27.
